# Effect of Long-Term Treatment with Alendronate on Bone Repair and Mineralization Around Implants in Rats

**DOI:** 10.1590/0103-644020235751

**Published:** 2024-12-06

**Authors:** Mario Henrique Arruda Verzola, Fausto Frizzera, Paulo Sergio Cerri, Ubirajara Pereira, Silvana Regina Perez Orrico, Rafael Scaf de Molon

**Affiliations:** 1 Department of Diagnosis and Surgery, School of Dentistry at Araraquara, Sao Paulo State University- UNESP. Araraquara, SP, 14801-930, Brazil; 2 Department of Odontology Sciences, Federal University of Espirito Santo- UFES, School of Dentistry, Vitória- ES, 29075-910, Brazil; 3 Department of Morphology, Genetics, Orthodontics and Pediatric Dentistry, Dental School, São Paulo State University- UNESP, Araraquara, SP, 14801-930, Brazil; 4 Institute of Chemistry, University of São Paulo - USP, São Carlos, SP, 13566-590, Brazil; 5 Advanced Research Center in Medicine, Union of the Colleges of the Great Lakes (UNILAGO), São José do Rio Preto15030-070, Brazil; 6 Department of Diagnosis and Surgery, School of Dentistry at Araçatuba, Sao Paulo State University - UNESP. Araçatuba, SP, 16015-050, Brazil

**Keywords:** alendronate, bisphosphonate, bone remodeling, dental implants, osseointegration, rats

## Abstract

The aim of this study was to evaluate the effects of long-term alendronate administration on bone repair and mineralization around osseointegrated implants in rats. A total of 160 female Wistar rats were randomly assigned to two groups: the control group (CTL) and the alendronate group (ALD). The ALD group received a subcutaneous injection of sodium alendronate (1 mg/kg/week), while the CTL group received weekly injections of saline solution. After 120 days of treatment, a bilateral implant was placed in the tibia of each rat. Ten rats from each group were euthanized at 5, 10, 15, 20, 25, 30, 45, or 60 days post-surgery. Picro-sirius red staining was utilized to assess the distribution and arrangement of collagen fibers near the implant threads. Bone mineralization mapping of the native bone adjacent to the implant was performed using images obtained through scanning electron microscopy (SEM) across all follow-up periods. SEM-based mineralization mapping revealed an increase in both the degree and homogeneity of bone mineralization in the ALD group compared to the CTL group. Alendronate administration affected collagen arrangement and distribution, leading to a connective tissue with reduced organization and thinner collagen fiber bundles. In conclusion, the findings demonstrated that alendronate administration resulted in a higher degree and homogeneity of bone mineralization, accompanied by reduced collagen content and organization, suggesting an impairment in bone remodeling around dental implants.

## Resumo

O objetivo deste estudo foi avaliar o uso prolongado do alendronato no reparo ósseo e na mineralização ao redor de implantes osseointegrados em ratos. Este estudo incluiu 160 ratas Wistar, distribuídas aleatoriamente em dois grupos: grupo controle (CTL) e grupo alendronato (ALD). As ratas do grupo ALD receberam injeção subcutânea de alendronato de sódio (1 mg/kg/semana), enquanto as do grupo CTL receberam injeção de solução salina uma vez por semana. Após 120 dias de tratamento, um implante foi colocado bilateralmente na tíbia dos animais. Dez ratas por grupo foram sacrificadas aos 5, 10, 15, 20, 25, 30, 45 ou 60 dias após a cirurgia. A coloração Picro-Sirius Red foi utilizada para avaliar a distribuição e disposição das fibras colágenas na área próxima às roscas do implante. O mapeamento da mineralização óssea no osso nativo adjacente ao implante foi realizado a partir de imagens adquiridas por microscopia eletrônica de varredura (MEV) em todos os períodos de acompanhamento. As análises das imagens de MEV demonstraram aumento no grau de mineralização óssea e maior homogeneidade no grupo ALD em comparação ao grupo CTL. A administração de alendronato interferiu no arranjo e no padrão de distribuição do colágeno, resultando em tecido conjuntivo com menor grau de organização e feixes de fibras colágenas mais finos. Em conclusão, os resultados deste estudo mostraram que a administração de alendronato levou ao aumento do grau e da homogeneidade da mineralização óssea e à redução do conteúdo e da organização do colágeno em comparação ao grupo CTL, sugerindo um comprometimento da remodelação óssea ao redor dos implantes dentários.

## Introduction

Alendronate, a nitrogen-containing bisphosphonate (BP), is extensively utilized in the treatment of skeletal diseases, such as osteoporosis (the most common chronic metabolic bone disease), bone metastases, Paget’s disease, and to reduce fragility fractures in postmenopausal women. BPs have a high affinity for hydroxyapatite, binding primarily to resorbing surfaces and acting as anti-catabolic agents. The primary function of BPs is to inhibit osteoclast activity, thereby reducing bone remodeling, subsequently increasing bone mineral density and content, and lessening osteoporosis-related morbidity ^1^. It has been hypothesized that alendronate affects different stages of bone remodeling, including microinjury repair, osteoclastogenesis, and osteogenesis, thus stimulating new bone formation by promoting the propagation and differentiation of osteoblasts while inhibiting osteoclast function ^2^. However, BPs inhibit the renewal of damaged bone and possess a half-life of roughly 10 years due to their permanent binding to the bone matrix ^3^ ^,^ ^4^, resulting in adverse effects such as osteonecrosis of the jaw (ONJ) ^5^ ^,^ ^6^.

Osteoporosis is a prevalent disorder that affects more than 200 million people worldwide. The economic impact of osteoporosis is substantial, leading to the loss of independence, pain, and disability in affected individuals. As the population continues to age and life expectancy rises, the incidence of osteoporosis is expected to increase. Consequently, the use of BPs is anticipated to rise in the coming years. Likewise, periodontal disease (PD) is a highly prevalent chronic inflammatory condition, associated with a dysbiotic biofilm in close contact with the gingival margin, resulting in the loss of periodontal ligament, root cementum, and alveolar bone. The severe form of PD is the sixth most prevalent chronic condition worldwide, with a prevalence of approximately 11.2%, affecting 734 million people ^7^. PD is considered the leading cause of tooth loss in adults. Consequently, with an aging population and longer lifespans, the replacement of missing teeth by dental implants is significantly increasing worldwide. Therefore, it seems reasonable to anticipate an increased number of BP users who are candidates for tooth replacement through implant-supported rehabilitation, and/or BP users with existing osseointegrated implants.

The effects of local or systemic administration of alendronate on osseointegration around dental implants have gained attention over the years due to potential adverse effects related to the inhibition of bone turnover in the alveolar bone, and/or because of the bone resorption inhibition that could lead to bone preservation and maintenance. In this context, previous studies have demonstrated that systemic administration of alendronate enhances the torque required for implant removal ^8^ ^,^ ^9^ ^,^ ^10^ and increases the amount of newly formed bone around osseointegrated implants in preclinical studies ^11^ ^,^ ^12^. We have previously demonstrated that long-term systemic use of alendronate in rats increased bone mineral density (BMD), enhanced fracture resistance, and improved osseointegration around dental implants ^13^. Importantly, numerous studies have investigated coating implant surfaces with alendronate, and the results of these studies showed improvements in newly formed bone, bone volume, and bone-to-implant contact (BIC), suggesting better osseointegration ^14^ ^,^ ^15^ ^,^ ^16^. Conversely, topical administration of alendronate into bone cavities resulted in impaired bone formation, reduced BIC, and weaker biomechanical fixation around implants ^17^ ^,^ ^18^. Additionally, systemic administration of alendronate has been shown to reduce bone turnover and compromise bone quality and quantity around dental implants in rats ^19^.

Taken together, the scientific literature remains divergent regarding the effects of systemic alendronate on osseointegration around dental implants. Therefore, the aim of this study was to evaluate the long-term effects of alendronate on bone repair and mineralization around osseointegrated implants in rats.

## Materials and Methods

### Animals

This study included 160 female Wistar rats (Rattus norvegicus albinus) aging 3 months old with mean body weight of 200-250g. The animals were housed in propylene cages, with controlled temperature (21°C) and humidity (65-70%), and a 12-h light-dark cycle. Animals consumed standard rat chow (Labina/Purina, Ribeirão Preto, Brazil) and received water ad libitum. The standard diet contained approximately 2.25 kcal/g and 22 g of protein, 48 g of carbohydrate, 4 g of total fat, 8 g of fiber, and 200 mg of sodium per 100 g of diet. The Ethical Committee for the use of animals in research approved this protocol (Proc. #16/2010), and the experimental study design followed all the ARRIVE guidelines.

### Study design

Rats were arbitrarily distributed in two groups using a table generated by the website Randomization.com (http://www.randomization.com): the control group (CTL) and the alendronate group (ALD). The rats in the ALD group received a subcutaneous (sc) injection of sodium alendronate (1 mg/kg/week), whereas the rats in the CTL group received a sc injection of saline solution once per week. After 120 days of drug administration, one implant was placed in each rat tibia. Ten rats per group were euthanized at 5, 10, 15, 20, 25, 30, 45, or 60 days after surgery, as previously described ^13^. The administration of sodium alendronate continued throughout the experimental periods. The rats weight was monitored weekly to adapt the ALD dosage.

### Implant Placement

Previous to the implant installation, rats were anesthetized with xylazine 2% (0.04ml / 100g bw - Rompum, Bayer S.A., São Paulo, Brazil) and ketamine 10% (0.08 ml / 100g bw - Rompum, Bayer S.A., São Paulo, Brazil). The surgical procedure was previously described ^13^ ^,^ ^20^. Briefly, to access the rat tibia, a single incision of nearly 10 mm was performed in both right and left tibiae. One implant site was prepared in the tibia proximal epiphysis using a low speed hand-piece. A titanium implant (4.0 x 2.2 mm; Conexao® Implant System, Brazil) with a machined surface was placed till the cortical bone using an electronic motor (BLM 600®; Driller Equipment, SP, Brazil). Later, the soft tissues were sutured in layers with 5.0 bioabsorbable (Vicryl, Ethicon®, Brazil) and 4.0 silk (Ethicon®, Brazil) sutures. The rats received a single intramuscular injection of a mixture of penicillin and streptomycin (0.1 ml/kg bw), and three intramuscular injections of 5 mg/kg corticoid (once per day).

After the follow-up periods, the rats were sacrificed by an overdose of anesthetics and the tibiae were removed and fixed in 4% paraphormoldehyde for 48 hours. Then, the tissue samples were used for evaluation of arrangement and distribution of collagen fibers by means of picro-sirius red staining and for scanning electron microscopy (SEM) to analyze the mineralization map.

### Histological Processing

The tibia containing the implants and the surrounding tissues were dehydrated in an increasing series of ethanol (60-100%), and then embedded in light-curing resin (Technovit 7200 VLC; Wehrheim, Germany), as described previously ^13^. Then, the tibiae were cut along the center of the implant, along its long axis, using a cutting- grinding unit (Exakt Cutting System, Wehrheim, Germany). One of these sections was ground and polished (Exakt Apparatebeau, Hamburg, Germany) to a final section thickness of approximately 30μm; Subsequently, they were stained with picro-sirius red for evaluation of collagen content, as described below.

### Picro-Sirius Red Staining

The slides were stained with 0.1% picro-sirius red solution for 1 hour to evaluate the distribution and arrangement of collagen fibers in the area of the implant threads and in the region adjacent to the implant. The slides were photographed with the aid of a polarized microscope (Olympus BX51) attached to a digital camera (Olympus DP71). Two polarizing filters (Olympus U_P110 and U_P115 Polarizing Filters) were coupled to the Olympus light microscope (model BX-51, Tokyo - Japan) for the analysis of picro-sirius stained sections. The image capture was obtained after standardization of light intensity, diaphragm opening, condenser height, and polarizer angle. The parameters considered for the analysis were: the presence of birefringent collagen and the arrangement and distribution of the collagen fibers in the region of the third thread of the implants. A blinded examiner (MHAV) in relation to the experimental groups performed the analysis.

### Scanning Electron Microscopy (SEM)

The implant sections that were not polished were metalized (Coating System BAL-TEC MED 020) and analyzed by scanning electron microscopy (ZEISS LEO 440, Cambridge, UK), as described elsewhere ^13^. Briefly, photomicrographs were achieved by using an OXFORD detector (model 7060 m; Cambridge, UK), operating with an electron beam at 20 kV. The images were taken with a backscattered electron beam using the four-quadrant backscattered detector (Electron Detector Type 400, UK).

### Mineralization Map

Mineralization content was evaluated using the images acquired by means of SEM (EDX Link Analytical, Isis System Series 200), using a detector SiLi Pentafet, ultrathin window ATW II (Atmosphere Thin Window), with a resolution of 133 eV at 5.9 keV and an area of 10 mm2. The images were achieved with a magnification of 160×, and the mapping of bone mineralization around the implants and native bone during the different periods of evaluation was performed. To this end, specific software was used (Scanning Probe Image Processor, Hørsholm- Denmark), which allows the bone mineralization mapping, by means of different shade of colors. On the scale available in the program, the colors close to blue represent areas with a lower degree of mineralization, while regions with colors close to red represent areas with greater degree of mineralization. A blinded examiner (MHAV) to the experimental groups performed a descriptive analysis regarding the distribution of different degrees of mineralization among the groups in all the follow-up periods.

### Data analysis

The data interpretation was performed by descriptive analysis evaluating the effects of alendronate administration on collagen content and mineralization in the different experimental time points.

## Results

The systemic administration of 1mg/kg/body weight of sodium alendronate in the rats did not result in any clinical intercurrence during the follow-up periods, and the rat’s body weight did not differ among the groups at the end of treatment (data not shown).

### Arrangement and Distribution of Collagen

The samples were stained with picro-sirius red and analyzed under a polarized light microscope. The analysis showed marked birefringence of the bone tissue, and the areas located in the third thread of the implants revealed differences in the arrangement of birefringent collagen between CTL and ALD groups during the experimental periods.

**Figure 1:**
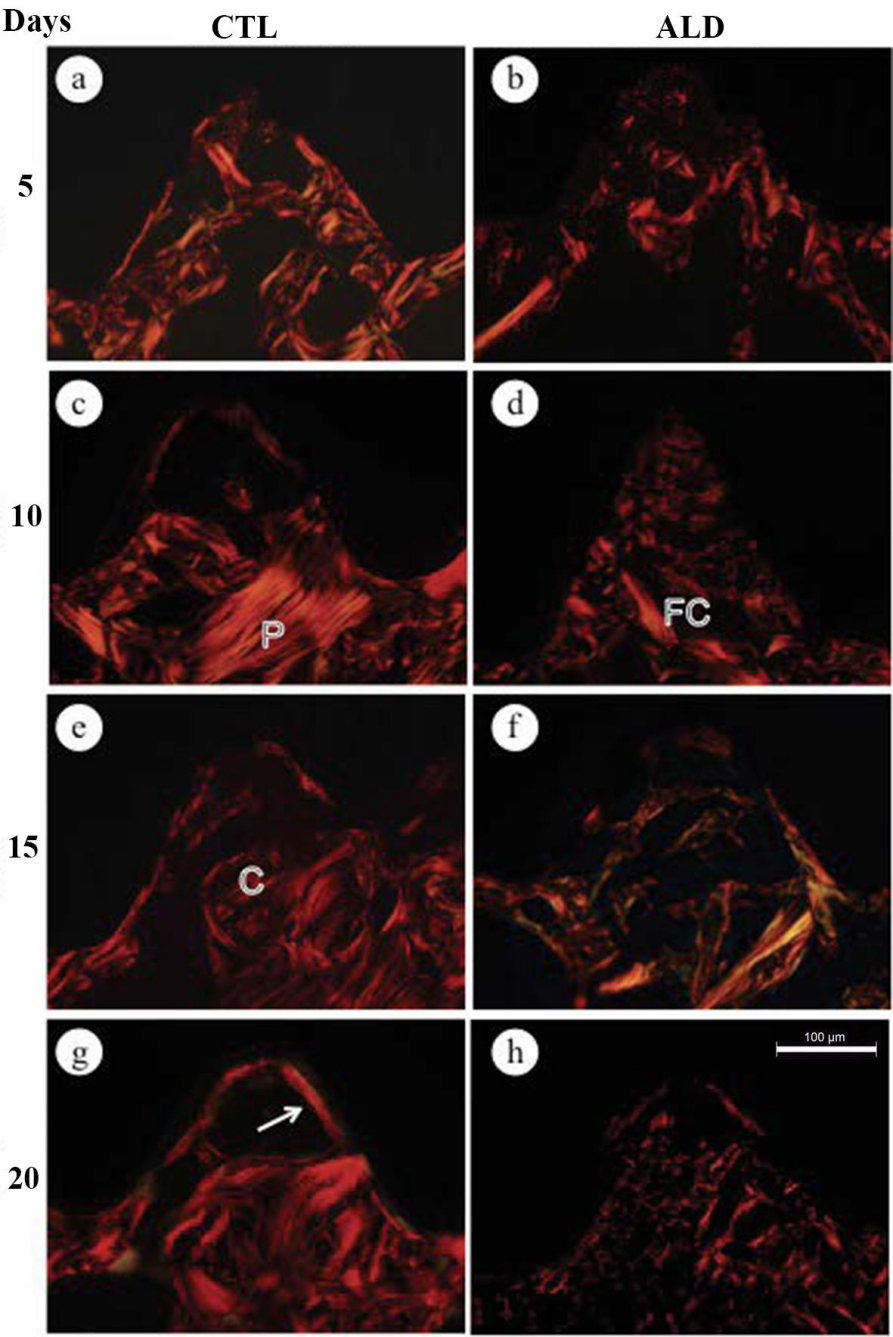
Photomicrographs of implant sections installed in the rat tibiae from CTL (a, c, e, g) and ALD (b, d, f, g) groups, stained with picro-sirius and analyzed under a light microscope with polarization filters. The area of the implant threads in the CTL group animals showed collagen fibers organized into concentric lamellae (C) and parallel lamellae (P), forming bone trabeculae. Note that in the ALD group, the bundles of birefringent collagen (FC) are irregularly arranged. A discontinuous layer of birefringent collagen (arrows), juxtaposed to the implant surface, was observed mainly from 20 days onward.

**Figure 2:**
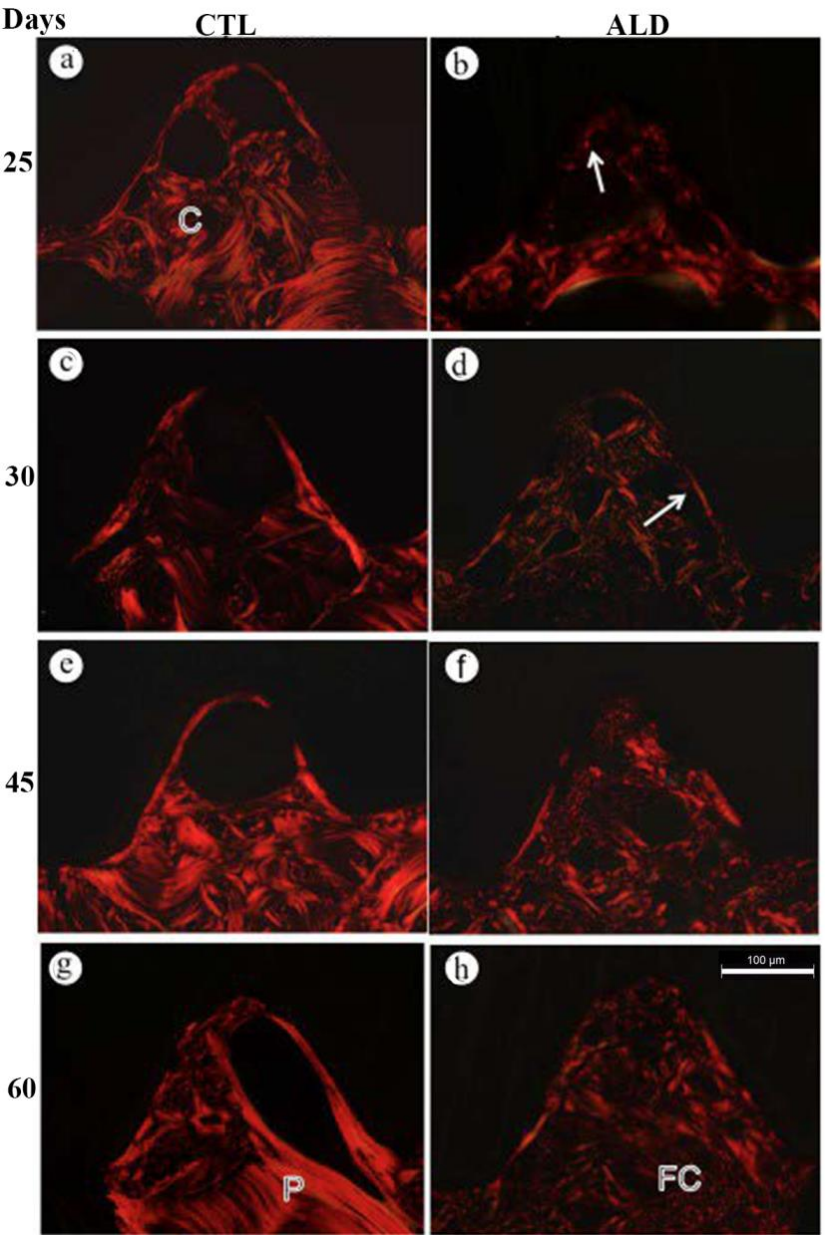
Photomicrographs of implant sections installed in the rat tibiae from CTL (a, c, e, g) and ALD (b, d, f, g) groups, stained with picro-sirius and analyzed under a light microscope with polarization filters. In the CTL group, the collagen bundles are organized in concentric (C) and parallel (P) lamellae, forming the trabecular bone that extends throughout the area of the implant threads. The presence of a birefringent collagen layer juxtaposed to the implant surface is evident in the CTL group. Note that in the ALD group, birefringent collagen bundles (FC) with an irregular arrangement are loosely distributed; birefringence is evidently lower in the ALD group compared to the CTL group. The discontinuous layer of birefringent collagen (arrows) juxtaposed to the implant surface is observed in the CTL group; in the ALD group, this layer (arrows) is thin.

In the control group at 5, 10, and 15 days irregular bone trabeculae showing thick collagen fiber bundles with marked birefringence were frequently observed in the areas close to the implant threads. Bone trabeculae exhibited collagen fiber bundles arranged in parallel layers or circles; some bone trabeculae containing irregularly disposed birefringent collagen fibers were located between the bone lamellae (Figure. 1A, C, E). At day 20, the trabeculae of lamellar bone extended throughout the implant thread. Furthermore, a thin layer of birefringent collagen fiber bundles was observed in intimate contact with the implant surface, in 57% of the analyzed threads (Figure. 1G). At day 25, the presence of this birefringent layer juxtaposed to the implants was observed in 70% of the evaluated areas (Figure. 2A). In the latter periods (30, 45 and 60 days), 100% of the areas presented with parallel lamellae layers of birefringent collagen; numerous of these lamellae were in continuity with the concentric collagen lamellae that filled the central portion of the implant threads (Figure. 2C, E, G). In the 45 and 60-days period, the collagen lamellae in a concentric arrangement, similar to the Havers system, were arranged between bone trabeculae containing collagen lamellae parallel arranged (Figure. 2E, G).

In the ALD group, at the initial periods, the thin and small bone trabeculae exhibited birefringent collagen forming a mesh with irregular arrangement. Thin bundles of birefringent collagen fibers were located between the irregular bone trabeculae (Figure. 1B, D, F). At 20 days, birefringent collagen bundles with irregular orientation were distributed throughout the implant thread. A birefringent layer juxtaposed to the surface of the implant was observed (Figure. 1H). Within 25 days, this birefringent layer juxtaposed with the surface of the implants, was observed in 50% of the areas, while at 30 days it was approximately 80% of the analyzed areas (Figure. 2B). In the 30, 45 and 60 days period, the bone trabeculae presented birefringent collagen fibers with an irregular arrangement and, frequently, the bundles of collagen were thin with little birefringence (Figures 2D, F, H).

### Mineralization Map

**Figure 3:**
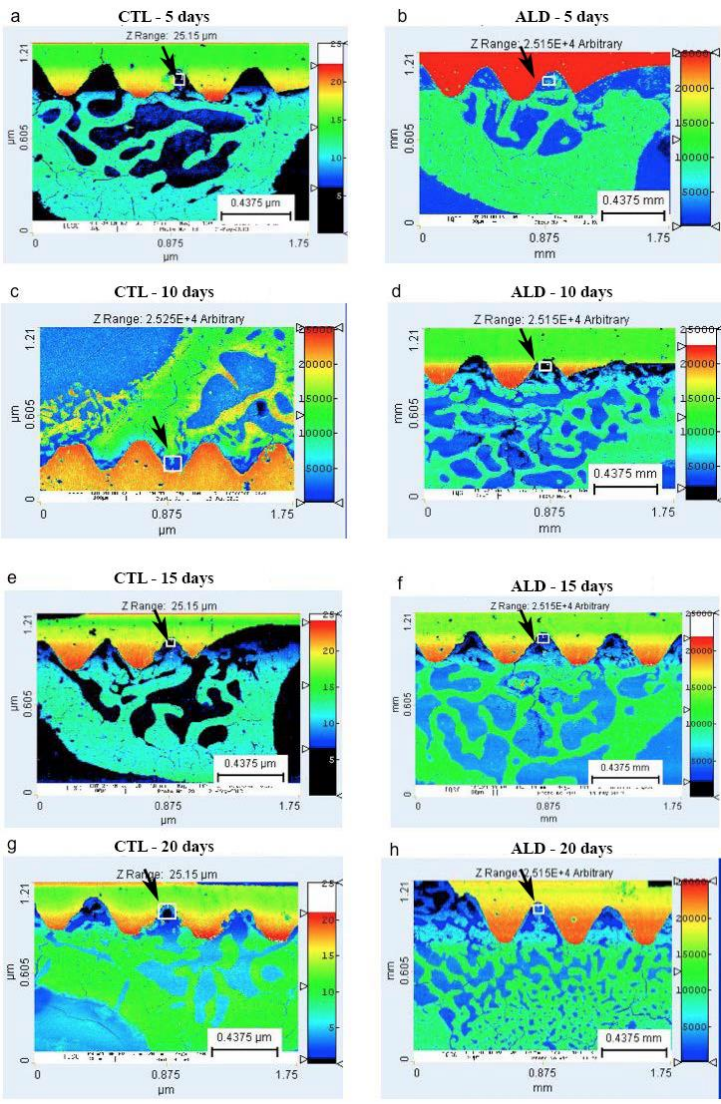
Mineralization mapping obtained through images acquired by scanning electron microscopy in the CTL and ALD groups at 5, 10, 15, and 20 days. The small squares and black arrows indicate the areas where the analyses were performed.

The CTL group demonstrated at 5 days period a small amount of bone tissue between the threads. In the region of the reminiscent bone, the predominance of light green color is observed, which is characteristic of mature bone tissue, with an intermediate degree of mineralization (Figure. 3A). For the ALD group at 5 days (Figure. 3B) a small amount of bone tissue is observed between the threads. The remaining bone presents a predominance of dark green color, representing higher degree of mineralization compared to the CTL group.

At the 10 days period, the CTL group (Figure. 3C) showed increase in bone tissue between the threads, although not completely filled. The reminiscent bone presented with a predominance of dark green color. The ALD group (Figure. 3D) showed an increase in the bone tissue between the threads although not fully completed.

The CTL group, in the 15 days period (Figure. 3E), showed bone tissue between the threads with a predominance of dark blue color, characteristic of less mineralized bone with empty spaces. For the ALD group (Figure. 3F), a greater space filling between the implant threads was observed, with predominance of light green color.

**Figure 4:**
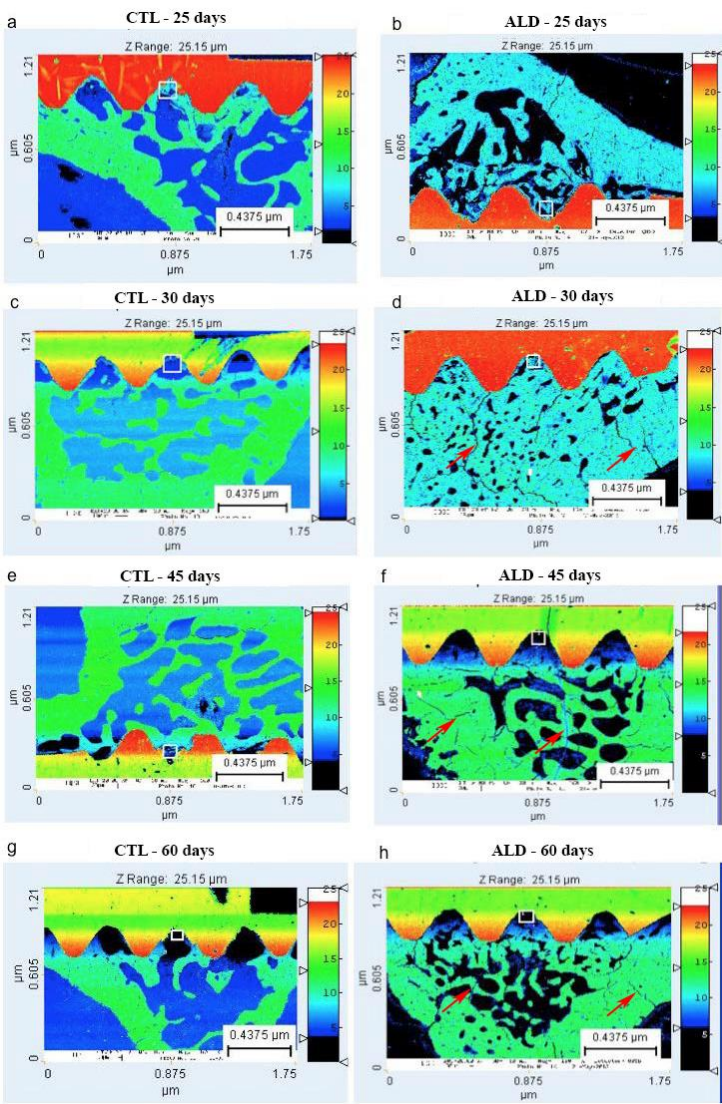
Mineralization mapping obtained through images acquired by scanning electron microscopy in the CTL and ALD groups at 25, 30, 45, and 60 days. The squares indicate the areas where the analyses were performed. The red arrows show areas of bone microcracks in the later periods of analysis.

At 20 days, the CTL group (Figure. 4G) showed bone tissue between the implant threads with a predominance of dark blue color characteristic of poorly mineralized tissue. The ALD group at 20 days (Figure. 3H), showed a greater amount of bone tissue between the threads, highlighted by the dark blue and light green colors, which characterize, respectively, the newly formed bone (still little mineralized) and reminiscent bone tissue with a more advanced mineralization degree.

The CTL group in the 25 days period (Figure. 4A) revealed greater bone filling between the implant threads with the presence of two different levels of mineralization, represented by dark blue and light green colors. In the region occupied by remaining bone tissue, blue and dark green colors were noted. As for the ALD group at 25 days (Figure. 4B), the spaces between the implant threads were filled by bone tissue with a predominance of light green. However areas without bone filling was also observed.

In the 30 days period, the CTL group (Figure. 4C) showed that the bone tissue between the implant threads have different levels of mineralization, represented by dark blue and light green colors, representing greater filling between the implant threads with less mineralized tissue. The ALD group (Figure. 4D) presented all the spaces between the implant threads filled by bone tissue, represented by the light green color, which was also observed in the region occupied by the remaining bone tissue.

At 45 days, the CTL group (Figure. 4E) showed bone tissue around the implant threads with different levels of mineralization, represented by the light green and dark blue colors, with a predominance of light green. For the ALD group (Figure. 4F) the bone tissue was observed between the implant threads with a predominance of the dark blue color representing less mineralized bone.

Finally, in the 60 days period, the CTL group (Figure. 4G) showed bone around the implant threads in light green and dark blue colors, although there are some spaces without bone filling. For the ALD group (Figure. 4H) it was observed in the area between the threads, a similar pattern to the control group regarding the degree of mineralization, although some areas have greater bone filling.

## Discussion

The present study investigated the long-term administration of alendronate on the mineralization process and the arrangement and distribution of collagen fibers in the bone tissue around implants placed in the rat tibia. Our results suggested that after four months of systemic alendronate administration, the process of bone remodeling was impaired mainly due to poor organization and distribution of collagen fiber bundles in the connective tissue and an increased degree and homogeneity of bone mineralization around the implant threads.

The chosen dosage of alendronate (1 mg/kg/weekly) by subcutaneous administration was based on the dose conversion of 70 mg once-weekly for a 70 kg human, calculated using the metabolic size measured by minimum energy cost and the metabolic rate, measured by the specific minimum energy cost per unit weight. Furthermore, we used the same dosage of alendronate in our previous studies, which involved the evaluation of dental implants ^13^ ^,^ ^19^ and the healing of critical size defects in the calvaria of rats ^20^ ^,^ ^21^. The 4-month period of alendronate administration was proposed to allow correlation with the long-term use in humans. According to Quinn R ^22^, 10.5 days of a rat's life corresponds to approximately one year in an adult human. Therefore, the 4-month alendronate administration might be considered equivalent to eleven years of treatment in humans.

In this study, we used mapping from the images obtained by the SEM method to evaluate the distribution of areas with greater or lesser degrees of mineralization. In the region of the remnant bone during the initial periods, the ALD group showed a higher degree of mineralization compared to the control group. This finding corroborates a previous study that showed an increase in bone mineral density and inhibition of bone resorption after alendronate treatment ^13^. Within 10 days after implant placement, the ALD group exhibited increased areas of mineralization with homogeneous characteristics compared to the CTL group, which might indicate impaired bone remodeling in treated animals. This observation aligns with a previous study ^23^ regarding the ability of BPs to influence bone remodeling, increasing bone mineral density. In the 15 and 20-day periods, the ALD group showed less mineralized newly formed bone around the implant and fewer empty spaces between the implant threads, suggesting a high rate of bone formation. In the region of native bone, ALD increased bone mineralization compared to the control group. A previous study ^24^ suggested that BPs could enhance not only osteoblastogenesis but also osteoblast activity, which might explain this finding.

During the late periods of analysis (25,30, 45, and 60 days), there was an increase in bone mineralization around the implant threads in the ALD group compared to the CTL group, with greater bone filling in some implant threads, paralleling observations made by Verzola et al. ^13^. Taken together, these data may help in understanding the possible causes of implant failures and the occurrence of osteonecrosis of the jaw (ONJ). According to Boivin et al. ^25^, a higher rate of mineralization, as observed in the ALD group, arises from the inactivity of the bone remodeling process, leading to increased secondary mineralization. One hypothesis for implant loss in BP patients is the impairment of the mechanical properties of bone tissue ^19^. Such deficiencies in the mechanical properties of bone may be related to the increase in bone mineral density (increased mineralization degree), possibly due to the reduction in the organic content (collagen) of the bone matrix ^25^. In addition, the reduction of the bone remodeling process decreases the natural repair of bone tissue, which may explain the occurrence of implant failures and the appearance of ONJ.

An interesting finding observed in the ALD-treated rats is the homogeneity of bone mineralization, represented by the strong green color (close to red), indicating inactivity of the remodeling process (higher mineralization). In light of this observation and considering that greater mineralization homogeneity may favor the accumulation and propagation of bone microcracks and microdamage ^26^ ^,^ ^27^ ^,^ ^28^ ^,^ ^29^ ^,^ ^30^ ^,^ ^31^, it seems reasonable to suggest that, up to a period of 25-60 days, the bone tissue in this region may be more prone to structural damage resulting from mechanical loads. These findings differed when compared to the vehicle-treated animals, which showed a high degree of bone heterogeneity. Furthermore, the increase in mineralization degree is closely related to a concomitant reduction in the organic content of bone, as indicated by the thin and less organized structure of the collagen fibers. Therefore, our data suggest a relationship between higher bone mineralization and diminished collagen fiber content. Taken together, our findings may aid in understanding the possible causes of implant failures and the occurrence of osteonecrosis of the jaw. However, it is important to bear in mind that our data is descriptive; thus, further studies should be conducted to uncover this association.

Collagen is one of the most abundant organic components in bone tissue, representing 90 to 98% of the organic matrix. Therefore, we sought to investigate the effects of long-term administration of ALD on the arrangement and distribution of collagen fiber bundles in the bone tissue near the implant threads. Overall, the CTL group presented a more organized distribution of collagen fibers, with greater thickness and a more structured arrangement compared to the ALD group. In the ALD group, the collagen bundles were thin and irregularly oriented. These findings demonstrate that, despite the drug's effect on the degree of mineralization, the constitution of the bone tissue collagen matrix was diminished. Altogether, although there was greater filling of the implant threads, the bone tissue formed in ALD-treated animals was characterized by weakened collagen content. According to a study conducted in postmenopausal women, after 2 and 3 years of ALN treatment, the distribution of the degree of mineralization in compact and cancellous bone showed a clear shift towards higher mineralization values and a decrease in the number of bone structural units with low mineralization values compared to the corresponding placebo group ^25^. This study demonstrated that the presence of a higher degree of mineralization in individuals treated with alendronate is closely related to the formation of a less organized organic matrix in smaller quantities ^25^. Our data, although not derived from osteoporotic animals, are consistent with the findings of Boivin et al. ^25^. Moreover, previous studies also showed that a higher degree of mineralization (bone homogeneity), combined with less organized and thin collagen fibers, increases the risk of developing microcracks ^27^ ^,^ ^30^ and microdamage to the bone in preclinical and clinical studies ^26^ ^,^ ^28^ ^,^ ^29^ ^,^ ^31^. These findings may directly interfere with the repair process, bone remodeling, and, consequently, a greater occurrence of failures.

We previously demonstrated that long-term treatment with sodium alendronate in rats led to positive effects on osseointegration, as evidenced by increased torque values for implant removal and increased BIC and bone area fraction occupancy ^13^. These results are likely attributed to reduced bone resorption with concomitant bone formation around the implant threads, which may have increased primary implant stability, thereby enhancing the removal torque values compared to the control group. Additionally, ALD also led to increased BMD, which would enhance implant osseointegration. However, the present study's results showed that the degree of mineralization was increased in the ALD group, and the organic component of bone tissue was impaired, suggesting a reduced capacity for bone remodeling. Therefore, it is crucial to consider that enhanced osseointegration in the periods up to 60 days in the ALD group might lead to an increased occurrence of failures in later periods. Consequently, more studies with long-term follow-up are recommended to investigate the deleterious effects of alendronate on bone around implants.

It is essential to note that our study has certain limitations that need to be considered. Firstly, we did not assess a group with osteoporotic animals. Given that alendronate is commonly used to treat osteoporosis in postmenopausal women, our results could have been more directly applicable had we used animals with compromised bone mineral density. Secondly, our data is descriptive and lacks quantification of the findings. While descriptive analyses are valid, they tend to be subjective. Hence, there is a need for further studies to conduct quantitative assessments of collagen content and to establish the correlation between reduced organic content and a higher degree of mineralization.

Collectively, our data demonstrated that long-term administration of alendronate increased bone mineralization and reduced collagen content quality. Therefore, the thin and irregularly oriented collagen organization, associated with more homogeneous bone mineralization, suggested a reduced capacity for bone remodeling, which might compromise the long-term success of osseointegration.
